# Photoprotective Effects of Cycloheterophyllin against UVA-Induced Damage and Oxidative Stress in Human Dermal Fibroblasts

**DOI:** 10.1371/journal.pone.0161767

**Published:** 2016-09-01

**Authors:** Cheng-Hua Huang, Hsin-Ju Li, Nan-Lin Wu, Chien-Yu Hsiao, Chun-Nan Lin, Hsun-Hsien Chang, Chi-Feng Hung

**Affiliations:** 1 School of Medicine, Fu Jen Catholic University, New Taipei City, Taiwan; 2 Department of Internal Medicine, Cathay General Hospital, Taipei, Taiwan; 3 Department of Chemistry, Fu Jen University, New Taipei City, Taiwan; 4 Department of Medicine, Mackay Medical College, New Taipei City, Taiwan; 5 Department of Dermatology, Mackay Memorial Hospital, Taipei, Taiwan; 6 Mackay Junior College of Medicine, Nursing, and Management, New Taipei City, Taiwan; 7 Department of Nutrition and Health Science, Chang Guang University of Science and Technology, Taoyuan, Taiwan; 8 Research Center for Industry of Human Ecology, Chang Gung University of Science and Technology, Taoyuan, Taiwan; 9 College of Pharmacy, Kaoshiung Medical University, Kaohsiung, Taiwan; 10 Biomedical Cybernetics Laboratory, Harvard Medical School, Boston, Massachusetts, United States of America; University of Alabama at Birmingham, UNITED STATES

## Abstract

Ultraviolet (UV) radiation, particularly ultraviolet A (UVA), is known to play a major role in photoaging of the human skin. Many studies have demonstrated that UV exposure causes the skin cells to generate reactive oxygen species and activates the mitogen-activated protein kinase (MAPK) pathway. Previous studies have also demonstrated that cycloheterophyllin has an antioxidant effect and can effectively scavenge free radicals. Extending the aforementioned investigations, in this study, human dermal fibroblasts were used to investigate the protective effect of cycloheterophyllin against UV-induced damage. We found that cycloheterophyllin not only significantly increased cell viability, but also attenuated the phosphorylation of MAPK after UVA exposure. Furthermore, cycloheterophyllin could reduce hydrogen peroxide (H_2_O_2_) generation and down-regulate H_2_O_2_-induced MAPK phosphorylation. In the *in vivo* studies, the topical application of cycloheterophyllin before UVA irradiation significantly decreased trans-epidermal water loss (TEWL), erythema, and blood flow rate. These results indicate that cycloheterophyllin is a photoprotective agent that inhibits UVA-induced oxidative stress and damage, and could be used in the research on and prevention of skin photoaging.

## Introduction

Ultraviolet (UV) irradiation is recognized to play a major role in photoaging and skin cancer [1, 2]. Many studies have indicated that UVA could be the most important contributor to numerous UV-induced alterations in the skin such as DNA/RNA damage [3], activation of mitogen-activated protein kinase (MAPK) signaling cascade [4–6], and production of reactive oxygen species (ROS) [7]. UVA is a significant oxidative stress inducer in the human skin, which results in photoaging and suppression of some immune functions [5].

**Fig 1:**
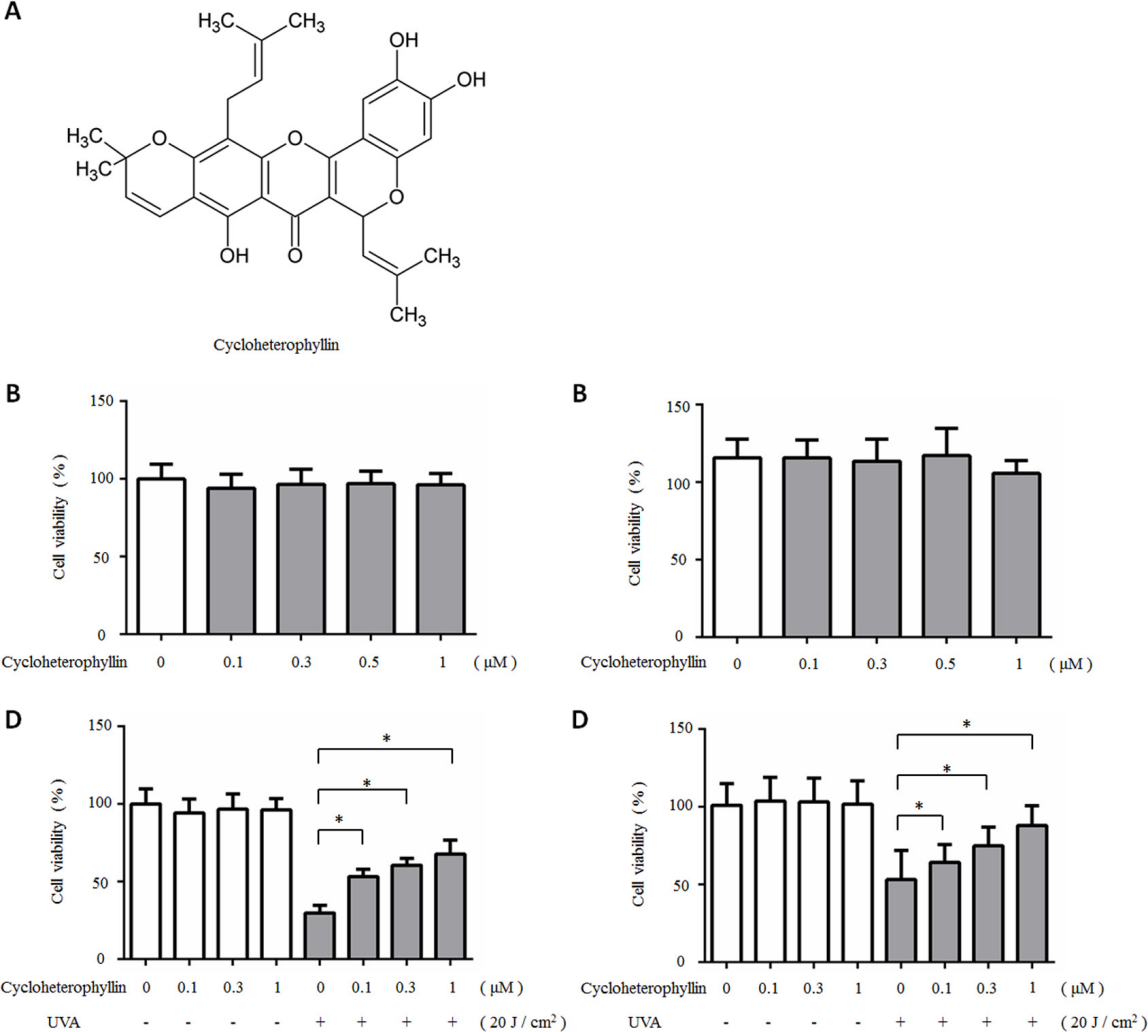
Cytotoxicity and protective effect of cycloheterophyllin on fibroblasts after UVA radiation exposure. The chemical structure of cycloheterophyllin. (B-C) The fibroblasts pretreated with indicated concentration of cycloheterophyllin (0.1–1 μmol/L) for 24 hours before UVA exposure. (D-E) Cell viability determined by MTT assay and Trypan blue assay 24 hours after UVA irradiation (20 J/cm^2^). Results are expressed as a percentage of control value and are represented by mean ± SD (n = 4). * *P* < 0.05 vs. UVA-exposed cells without cycloheterophyllin pretreatment.

Cycloheterophyllin (C_30_H_30_O_7_), a prenylflavone (Fig 1A), is isolated from *Artocarpus heterophyllus*, and has pharmacological and biological functions including anti-inflammatory effects, anti-platelet activities, and antioxidant properties [8–11]. However, it is unknown whether cycloheterophyllin can modulate the signaling pathways triggered by UVA in human dermal fibroblasts. Therefore, we investigated the underlying protective effect of cycloheterophyllin on UVA-induced damage in the fibroblasts, including the effects on UVA-induced generation of ROS generation and MAPK signaling. The data generated by this study can assist in unraveling the molecular mechanisms underlying the photoprotective effects of cycloheterophyllin against UVA-induced damage in dermal fibroblasts [12].

## Materials and Methods

### Ethics Statement

Foreskins were provided by the Mackay Memorial Hospital to the Fu Jen Catholic University with a notice stating an exemption from the institutional review board (IRB) (C10018), as no interaction occurred with the subjects and no identifiable information was made available to the researchers. All animal tests were approved by the Fu Jen Catholic University’s Institutional Animal Care and Use Committee (IACUC) policy.

### Materials

Cycloheterophyllin was isolated from the plant *Artocarpus heterophyllus* Lam., as described previously [9], and was dissolved in DMSO.

3-(4,5-Dimethylthiazol-2-yl)-2ami,5-diphenyltetrazolium bromide (MTT), aprotinin, leupeptin, phenylmethylsulfonyl fluoride (PMSF), sodium fluoride (NaF), and sodium orthovanadate were purchased from Sigma Chemical Co. (St Louis, MO).

Primary antibodies anti-p38 and anti-phospho-JNK were purchased from Cell Signaling Technology (Beverly, MA); anti-JNK, anti-ERK1/2, and anti-phospho-p38 were purchased from R&D System, Inc. (Minneapolis, MN); anti-phospho-ERK1/2 was purchased from Santa Cruz Biotechnology (Santa Cruz, CA). The secondary antibodies, anti-rabbit-HRP, and anti-goat-HRP were also purchased from Santa Cruz Biotechnology (Santa Cruz, CA). The antibody 2'-7'-dichlorofluorescein diacetate (DCFDA) was purchased from Invitrogen Technologies (Carlsbad, CA).

### Cell cultures

The human primary fibroblasts were isolated from neonatal foreskins. The foreskins were provided by the Mackay Memorial Hospital to Fu Jen Catholic University with a notice stating an exemption from the IRB (C10018), as no interaction occurred with subjects and no identifiable information was made available to the researchers. The cells were cultured in a humidified incubator at 37°C with 5% CO_2_. For most of the experiments, cells reaching 90%–95% confluence were starved and synchronized in serum-free DMEM for 24 hours before being subjected to further analysis.

### Drug treatment and UVA irradiation

Fibroblast cells were cultured on 24-well plates, 6-well plates (Costar, Cambridge, MA), and in 6-cm culture dishes (Costar, Cambridge, MA) for the cell viability assays, flow cytometric analysis, and Western blot analysis, respectively. Next, the cells were pretreated with various concentrations of cycloheterophyllin for 24 hours. After two washes with DMEM, the cells were incubated with 300 μl/well, 500 μl/well, and 1000 μl/dish phosphate-buffered saline (PBS), under UVA irradiation in different trials. The UVA irradiation was performed with a Bio-Sun system illuminator from VL (Vilber Lourmat, France) immediately after the DMEM-washes, as suggested by the manufacturer. The UVA lamps in the illuminator emit ultraviolet rays between 355 and 375 nm, with peak luminosity at 365 nm. UVA radiation was supplied by a closely spaced array of four UVA lamps, which delivered uniform irradiation at a distance of 10 cm. To obtain a UVA irradiation dose of 20 J/cm^2^, it took approximately 74–80 minutes (Irradiance: 4.2–4.5 mW/cm^2^). Based on a programmable microprocessor, the Bio-Sun system constantly monitored the UV light emission. The irradiation stopped automatically when the energy received matched the programmed energy (range of measure: 0–9999 J/cm^2^). After UVA exposure, the cells were fed fresh DMEM-containing cycloheterophyllin and incubated for an additional amount of time, as specified in the following section, before being collected for further analysis.

### Cell viability assays

The viability of cells was determined by MTT assay. The MTT assay has been previously described [13]. Briefly, cells pretreated with PBS or cycloheterophyllin were exposed to UVA and incubated for an additional 24 hours. After a brief wash with PBS, MTT (0.5 mg/ml in DMEM) was used for the quantification of living metabolically active cells. Mitochondrial dehydrogenases metabolized MTT to a purple formazan dye, which was analyzed photometrically at 550 nm. Cell viability is proportional to the absorbance measured.

The trypan blue exclusion method was used to accurately determine cell viability subsequent to isolation, according to the manufacturer’s protocols. Human dermal fibroblasts were seeded on a 35-mm dish (Corning). After reaching subconfluency, the fibroblast medium was renewed and the cells were subjected to 24-hour pre-treatment with cycloheterophyllin. The cells were collected from their cellular suspension using Trypsin-EDTA (Gibco BRL/Invitrogen, Carlsbad, CA) and stained with an equal volume of 0.4% trypan blue dye for 1 min. The cells were counted using a dual-chamber hemocytometer and a light microscope.

### Flow cytometric analysis of intracellular ROS

Intracellular production of ROS was assayed as previously described [14], with minor modifications. Confluent fibroblasts starved with serum-free DMEM were pre-treated with various concentrations of cycloheterophyllin for 24 hours. Afterwards, the cells were washed with PBS and DMEM, and then treated with 5 μg/ml 2'-7'-dichlorofluorescein diacetate (DCFDA) in DMEM for 30 minutes. After another brief wash, the cells were irradiated with UVA and were collected by scraping and centrifugation. The cell pellets were resuspended in 1 ml PBS and analyzed immediately by the Partec CyFlow ML flow cytometer (Partech GmBH, Munster, Germany) at excitation and emission wavelengths of 488 and 525 nm, respectively. Fluorescence signals of 10,000 cells were collected to calculate the mean fluorescence intensity.

### Cell lysate preparation and Western blot analysis of JNK, ERK, and p38

Fibroblast cells treated with or without UVA irradiation were washed with PBS twice. They were then lysed in radioimmunoprecipitation assay buffer [17 mM Tris–HCl, pH 7.4, 50 mM NaCl, 5 mM EDTA, 1 mM sodium fluoride, 1% Triton X-100, 1% sodium deoxycholate, 0.1% SDS, 1 mM sodium orthovanadate, 1 mM PMSF, and 1 μg/ml aprotinin and leupeptin (freshly prepared)]. After sonication, the lysate was centrifuged (14,000 *g* for 10 min at 4°C), and the supernatant was removed. The protein content was quantified by a Pierce protein assay kit (Pierce, Rockford, IL). Total protein was separated by electrophoresis on 8% SDS–polyacrylamide gels. The proteins were then electroblotted onto PVDF membranes and probed using the indicated specific antibodies. Immunoblots were detected by enhanced chemiluminescence (Chemiluminescence Reagent Plus from NEN, Boston, MA). For some of the experiments, the PVDF membrane was stripped at 60°C for 30 minutes with a stripping buffer (62.5 mM Tris-HCl, pH 6.7, 2% SDS, and 100 mM β-mercaptoethanol).

### In Vivo Bioengineering Evaluations

Eight-week-old male BALB/c mice were obtained from the National Laboratory Animal Center, Taipei, Taiwan. Animals were housed and handled according to institutional guidelines. Briefly, mice were housed one per cage with controlled temperature (21–25°C), humidity (60±5%), and light (12/12 h light/dark cycle) for one week. Alfalfa-free food (5058, LabDiet, Framingham, MA) and water were administered *ad libitum*. All of the animal experimental protocols were reviewed by the committee and conducted after obtaining the Affidavit of Approval of Animal Use Protocol of the Fu Jen Catholic University. The dorsal skin of the BALB/c mice was then treated with different concentrations of cycloheterophyllin before 8 J/cm^2^ UVA irradiation for seven days. TEWL, erythema, skin hydration, and blood flow were assessed every day with MPA-580 (Courage & Khazaka, Cologne, Germany) and FLO-N1 (Omegawave, Tokyo, Japan) before UVA irradiation. The surface changes in the dorsal skin were recorded by photography. When the animals’ suffering clearly negated the need to continue with the experiment, they were dropped from the study and euthanized by CO_2_ in accordance with the Fu Jen Catholic University’s Institutional Animal Care and Use Committee (IACUC) policy. This study was reviewed and approved by the Fu Jen Catholic University’s IACUC

### Statistical analysis

Unless otherwise indicated, data are expressed as mean ± standard deviation (SD) by using the GraphPad Prism Program 6 software (GraphPad Software San Diego, CA). Comparison of the mean survival rates of cells under UVA irradiation with and without cycloheterophyllin was made using one-way ANOVA followed by Dunnett’s t-test for multiple comparisons. We considered p < 0.05 to be statistically significant.

## Results

### Cycloheterophyllin protected the fibroblasts from UVA-induced cell death

We first used an MTT assay to evaluate whether cycloheterophyllin would induce cytotoxicity in the fibroblasts, and our results showed that cycloheterophyllin (0.1–1 μM) exhibited low cytotoxicity after 24 hours of treatment (Fig 1B). The effects of UVA irradiation were also determined by the MTT test. Following another period of UVA (10–30 J/cm^2^) exposure for 24 hours, we observed a dose-dependent decrease in cell viability (data not shown). We chose UVA 20 J/cm^2^ to perform further experiments. To investigate the photoprotective effects of cycloheterophyllin, fibroblasts were pretreated with different doses of cycloheterophyllin. Results showed that cycloheterophyllin increased cell viability after UVA irradiation (20 J/cm^2^) in a dose-dependent manner (Fig 1B).

### Cycloheterophyllin inhibited UVA-induced MAPK activation in the human dermal fibroblasts

**Fig 2:**
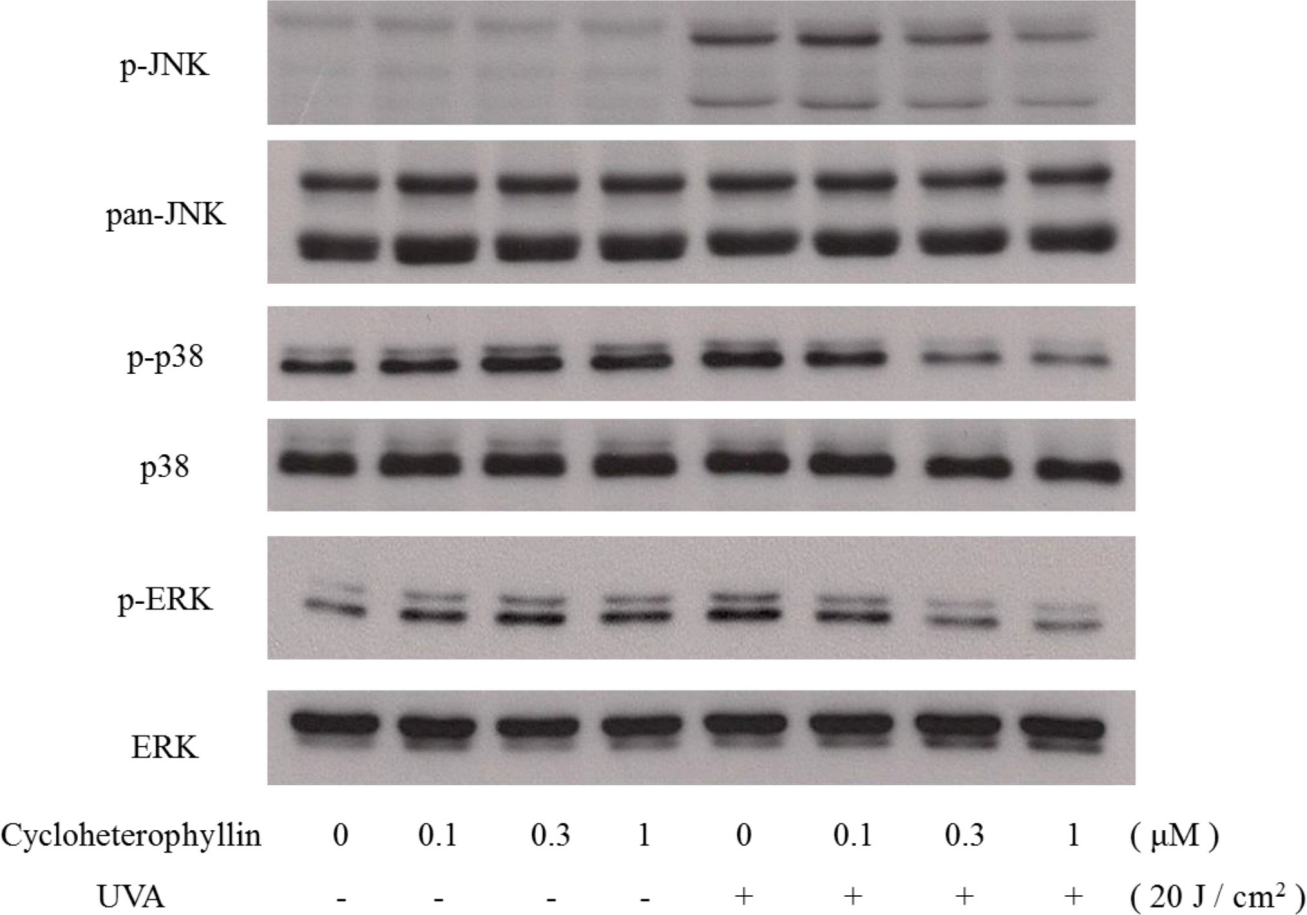
Cycloheterophyllin inhibited UVA-induced MAPK activation in human dermal fibroblasts. Western blotting was used to evaluate the changes in the phosphorylated ERK1/2, p38, and JNK expressions after UV irradiation. The human dermal fibroblasts were pretreated with different doses of cycloheterophyllin and irradiated with UVA. After irradiation, cell lysates were harvested for analysis.

MAPK signaling is critically involved in UV-activated pathways and is known to regulate various downstream effects in the skin cells [13, 15–17]. Subsequent to the evaluation of the effects of cycloheterophyllin on the UV-irradiated dermal fibroblasts described previously in this study, we investigated the possible signaling pathways modulated by cycloheterophyllin. As shown in Fig 2, UVA irradiation induced phosphorylation of ERK1/2, p38, and JNK. Cycloheterophyllin pretreatment inhibited JNK phosphorylation in a dose-dependent manner; it also significantly decreased p38 phosphorylation and attenuated ERK1/2 activation.

### Cycloheterophyllin reduced H_2_O_2_-induced cell death in the human dermal fibroblasts

**Fig 3:**
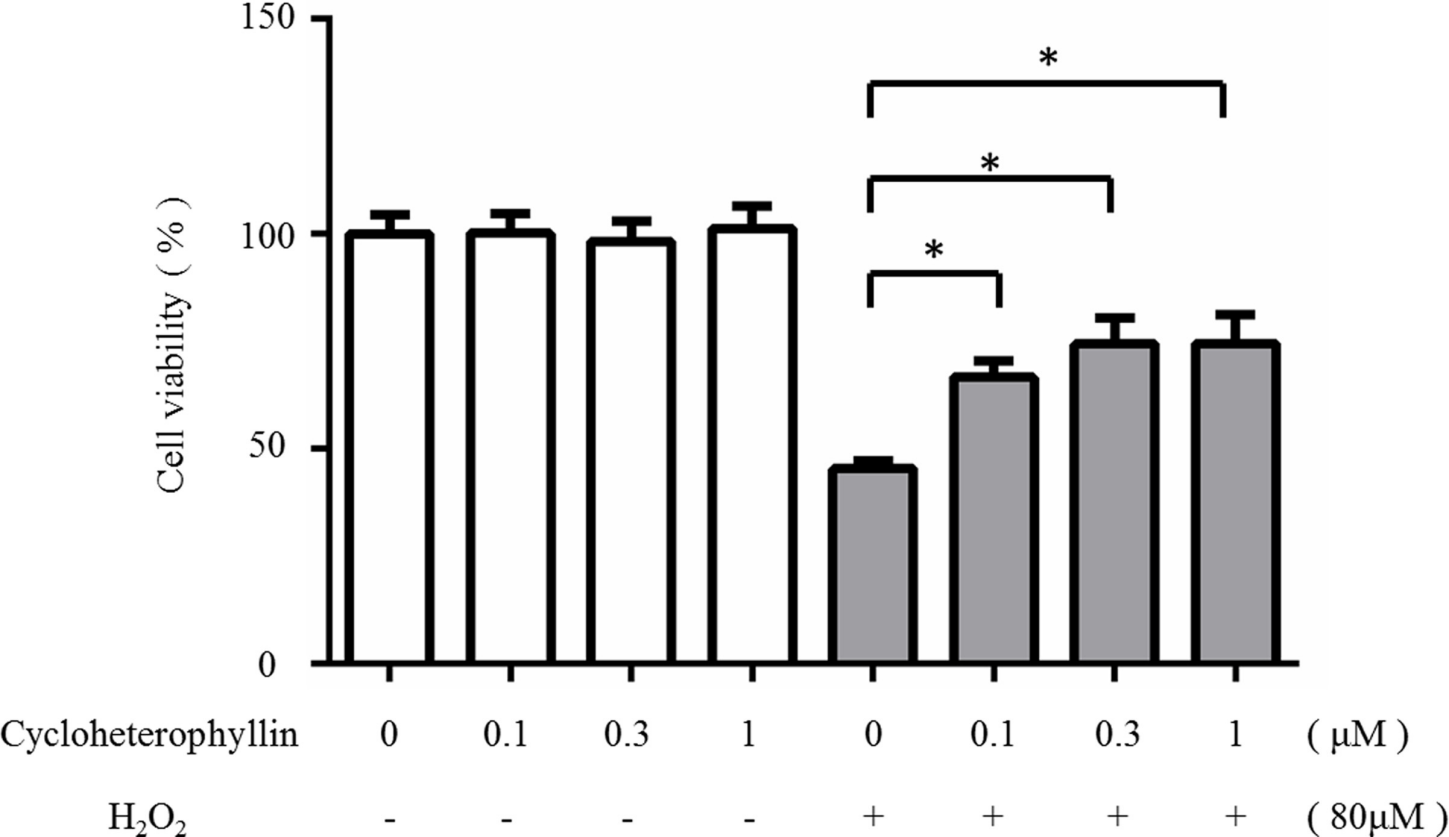
Cycloheterophyllin ameliorated the decreased viability of human dermal fibroblasts induced by H_2_O_2_. The fibroblasts were pretreated with cycloheterophyllin (0.1–1 μM). Cell viability was determined by MTT assay after H_2_O_2_ treatment. Results are expressed as a percentage of control value and are represented by mean ± SD (n = 4). *, *P* < 0.05 vs. H_2_O_2_-treated cells without cycloheterophyllin pretreatment.

Treating the human skin fibroblasts with 80 μM of H_2_O_2_ induced a decrease in viability when compared to that of the control. Pretreating the cells with 1 μM cycloheterophyllin prior to the H_2_O_2_ treatment increased the cell survival rate, which suggests that cycloheterophyllin suppresses the H_2_O_2_-induced cytotoxicity (Fig 3).

### Cycloheterophyllin inhibited UVA-induced intracellular ROS production in the human dermal fibroblasts

**Fig 4:**
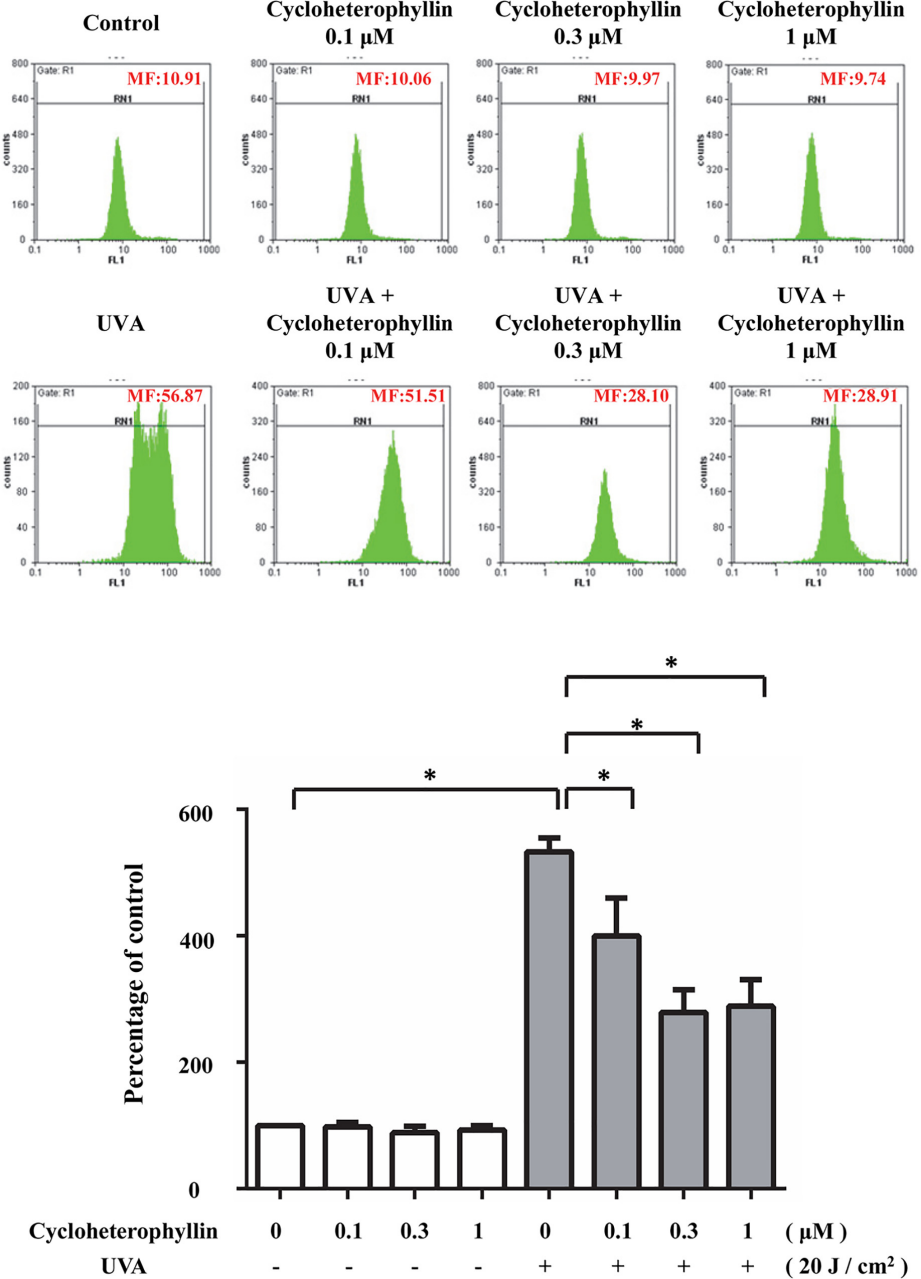
Cycloheterophyllin inhibited UVA-induced intracellular ROS production in human dermal fibroblasts. Flow cytometry was used to investigate ROS production in human dermal fibroblasts after UV irradiation. After the pretreatment with cycloheterophyllin at the indicated concentrations, cells were exposed to UVA. Data are expressed as a percentage of the control value (mean ± SD, n = 3). *, p < 0.05 vs. irradiated cells without drug treatment.

UVA-induced oxidative stress has various harmful effects [18–20]. In this study, cycloheterophyllin showed ROS scavenging ability. As shown in Fig 4, increased ROS production in the primary dermal fibroblasts after UVA irradiation was detected by flow cytometry, and it was significantly suppressed by pretreating the cells with cycloheterophyllin.

### Cycloheterophyllin inhibited H_2_O_2_-induced MAPK activation in the human dermal fibroblasts

**Fig 5:**
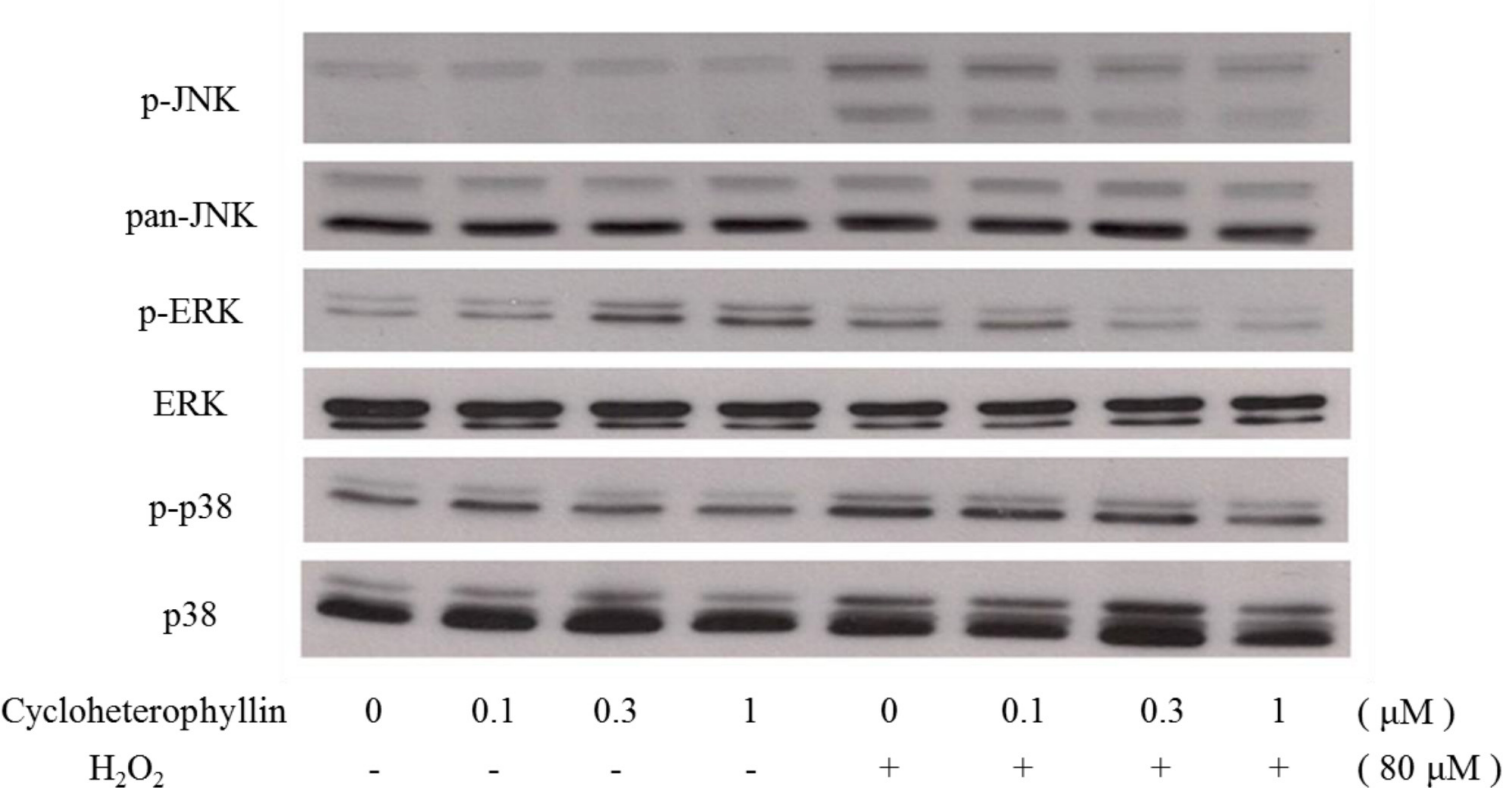
Cycloheterophyllin inhibited H_2_O_2_-induced MAPK activation in human dermal fibroblasts. Western blotting was used to evaluate the changes in phosphorylated ERK1/2, p38, and JNK expression after H_2_O_2_-treatment. Human dermal fibroblasts were pretreated with different doses of cycloheterophyllin, followed by treatment with H_2_O_2_. After treatment, cell lysates were harvested for analysis.

MAPK signaling is critically involved in H_2_O_2_-activated pathways and regulates various downstream effects in the skin cells [21, 22]. Subsequent to the evaluation of the effects of cycloheterophyllin on UV-irradiated dermal fibroblasts described earlier, in this study, the possible signaling pathways modulated by cycloheterophyllin were investigated. As shown in Fig 5, H_2_O_2_ induced ERK1/2, p38, and JNK phosphorylation. Pretreatment with cycloheterophyllin inhibited JNK phosphorylation in a dose-dependent manner, significantly decreased p38 phosphorylation, and attenuated ERK1/2 activation.

### Cycloheterophyllin improved UVA-induced skin lesions in vivo

**Fig 6:**
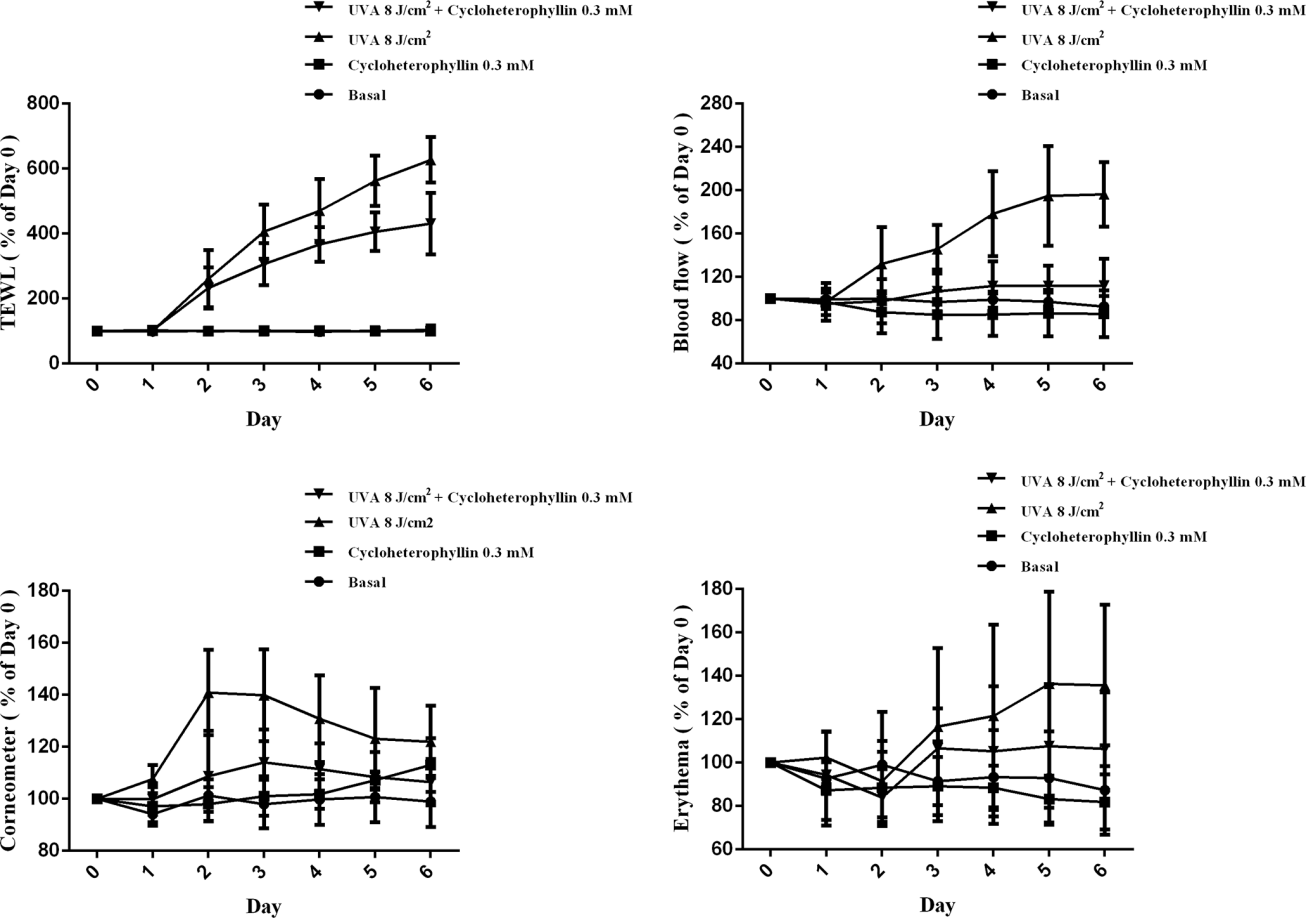
In the *in vivo* study, cycloheterophyllin can alleviate UVA-induced skin lesions. The physiological parameters of the skin surface, such as trans-epidermal water loss (TEWL), erythema, skin hydration, and rate of blood flow, changed after treatment with cycloheterophyllin. Data are expressed as a percentage of the day 0 values (mean ± SD, n = 5).

We first set up an animal model of skin barrier damage by applying UV irradiation daily on the dorsum of BALB/c mice for seven days. We then assessed the trans-epidermal water loss (TEWL), erythema, blood flow rate. To evaluate the *in vivo* effect of cycloheterophyllin, we pretreated the mice with cycloheterophyllin before the UV exposure and investigated the changes in the morphologies and biological parameters 24 hours later. The topical application of cycloheterophyllin before UVA irradiation significantly decreased TEWL, erythema, and blood flow rate (Fig 6). These results indicate that cycloheterophyllin efficiently inhibited UVA-induced damage, suggesting its beneficial role in the prevention of skin photoaging.

## Discussion

Cycloheterophyllin is one of the prenylflavones isolated from the root bark of *A*. *heterophyllus*. Previous studies have reported that cycloheterophyllin has anti-platelet, anti-oxidative, anti-inflammatory, anti-diabetic, and anti-tuberculosis activity [9–11, 23, 24]. In this study, we further investigated the molecular mechanisms and biological activities of cycloheterophyllin with a particular focus on its protective effect against UVA-induced damage and oxidative stress injury. We found that, at a low concentration, cycloheterophyllin could effectively inhibit UVA-induced damage of the fibroblasts; in the nanomolar (nM) concentration range, it can effectively reduce the UVA-induced damage to cell viability.

Previous studies noted that the main factor in the UVA-induced damage to the skin cells is the presence of ROS, including hydrogen peroxide; singlet oxygen; and hydroxyl radicals, which affect the activation of the downstream cellular signaling pathways and cause damage to the skin. In this study, cycloheterophyllin inhibited UVA- and ROS-induced phosphorylation of the MAPK pathway (JNK, ERK, and p38) in a dose-dependent manner. For these known signaling pathways, we also investigated the generation of ROS to explore the antioxidant and photoprotective effects of cycloheterophyllin treatment. We pretreated the fibroblasts with cycloheterophyllin to find that cycloheterophyllin effectively reduces the amount of ROS after UVA exposure and exhibits antioxidant capacity. However, we identified that cycloheterophyllin has a protective effect only on UVA-induced damage and not on UVB-induced damage, which causes inflammation of the skin [25, 26]. The difference could be attributed to the differential anti-inflammatory and antioxidant pharmacological effects of cycloheterophyllin on the skin cells; however, this requires further study. In recent years, a growing number of studies have reported that the skin neuroendocrine system plays an important role in sensing the environment, and its components regulate local and global homeostasis following the algorithms of classical neuroendocrine or endocrine systems [27]. The results described above suggest that the neuroendocrine system influences the physiological responses of the skin. This information will be a significant breakthrough in our future study of skin photoaging.

Many studies on the protective effects of natural plant ingredients against UV and oxidative stress damage have been reported. For example, silymarin has hepatoprotective capabilities and catechin, which is abundantly present in green tea as (-)epigallocatechin gallate (EGCG) and (-)epicatechin gallate (ECG), possesses photoprotective effects against both UVA- and UVB-induced damage [14, 28, 29]. Recently, our studies showed that pretreatment with chrysin can effectively protect against UV-induced damage and even reduce cell apoptosis, ROS generation, and cyclooxygenase 2 (COX-2) expression [30]. Furthermore, chrysin reduces UVA-induced matrix metal proteinase-1 (MMP-1) upregulation [31]. In addition, previous research have reported that melatonin can be considered a protective agent, a survival factor with anti-genotoxic properties, or even a protector of genomes against UV radiation-induced cellular transformation including skin cancers and skin aging [32]. Cycloheterophyllin is one of the flavonoids; therefore, it may have the same effect on cyclooxygenase expression and matrix metalloproteinase release.

In general, there are two types of melanin in the human skin: eumelanin and pheomelanin. The former is more abundant in humans, is a brownish-black pigment, and is present mainly in the skin and hair. The latter is a reddish yellow pigment [33, 34]. Natural melanin has complex functions, including the capability of absorbing, scattering, and reflecting light at different wavelengths, and it may protect the skin from UV-induced damage [35, 36]. The human skin activates tyrosinase in the melanocytes after UV radiation exposed, which triggers the conversion of tyrosine to melanin to protect the skin against UV-induced damage [34, 36]. For the skin, melanin not only plays a role in photoprotection and uses its physico-chemical properties to avoid the harmful effects of UV radiation [33, 37], but also neutralizes UV-induced free radicals produced in the skin [36, 38]. In this study, we did not investigate the effects of and changes in melanin after the cycloheterophyllin and UV treatment. However, because melanin plays a role in photoprotection from UV-induced damage, future studies should examine the effects of melanin.

Normal murine hair growth is cyclic, and the major stages can be classified into hair follicle growth (anagen), regression (catagen), and quiescence (telogen) [39–42]. In anagen, hair follicle cells differentiate into new cells and hair grows rapidly. In catagen, hair follicle cells stop dividing and progress into a period of dormancy, but hair will continue to grow. Progress from catagen to telogen manifests itself with apoptotic death of a large number of follicle cells, whereupon hair follicle enters telogen [43, 44]. If moderately stimulated with growth factors, hormones, or drugs, hair follicle cells will pass to the next round of growth cycle [45–47]. The first and easiest to measure, among various parameters of hair follicle stage classification, is its length. The hair follicle length is the distance from the dermal papilla to the epidermis. In anagen, the hair follicle reaches its maximal length, while the minimal length—in telogen. Therefore, the thickness and pigmentation of the skin in mice is associated with stage-dependent changes of hair follicle cycle [42]. In this study, we removed mice hair by shaving before the experiment, which does not induce anagen. However, the progress in hair cycle and its influence on the effects observed in vivo should be considered, and we intend to extend our future studies by the effects of the hair cycle.

The skin is a metabolically active barrier; a previous study reports that a metabolic barrier by cytochrome P450 (CYPs) can regulate the homeostasis of a metabolic barrier through activation or inactivation of biologically relevant molecules [48, 49]. The local steroidogenic system is composed of locally expressed CYPs, which may be a potential treatment for prevention and attenuation of tumor progression in certain skin cancers [50]. The major barrier to water loss from the skin is the stratum corneum. Keratinocytes are located below the stratum corneum, and express AQP-3 in their plasma membranes. AQP-3 increases the permeability of the stratum corneum to water, urea, and glycerol, thus hydrating the skin to normal levels [51]. In our study, we found that cycloheterophyllin pretreatment could attenuate skin water loss after UV exposure and decrease TEWL. This phenomenon suggests that cycloheterophyllin may play a key role in AQP-3 expression. It was reported that the H_2_O_2_-induced pathway would cause a downregulation of AQP-3 [52]. Cycloheterophyllin was shown to scavenge ROS in this study; therefore, we speculate that cycloheterophyllin may have the ability to help AQP-3 protein recovery in skin cells. In the *in vivo* experiments, applying cycloheterophyllin alone did not cause skin irritation, but significantly ameliorated UVA-induced skin peeling on the backs of the mice. In addition, we found that cycloheterophyllin could effectively reduce TEWL after UVA irradiation. TEWL measurements can be used as an indicator for the internal barrier properties of skin; a higher value indicates a poorer water-holding capacity of the stratum corneum. Because the skin structure deteriorates after UVA exposure, TEWL values increase significantly, and the cycloheterophyllin-pretreated group exhibited reduction in TEWL values. Cycloheterophyllin was also found to decrease UVA-induced skin redness and inflammation. These results may lay the foundation for clinical anti-photoaging treatments in the future.
